# The N-Terminal Domain of ERK1 Accounts for the Functional Differences with ERK2

**DOI:** 10.1371/journal.pone.0003873

**Published:** 2008-12-04

**Authors:** Matilde Marchi, Angela D'Antoni, Ivan Formentini, Riccardo Parra, Riccardo Brambilla, Gian Michele Ratto, Mario Costa

**Affiliations:** 1 Institute of Neuroscience CNR, Pisa, Italy; 2 NEST-INFM, Scuola Normale Superiore, Pisa, Italy; 3 San Raffele Scientific Institute, Milano, Italy; 4 Scuola Normale Superiore, Pisa, Italy; 5 Italian Institute of Technology (IIT), Pisa, Italy; 6 Università degli Studi di Milano, Milano, Italy; University of Edinburgh, United Kingdom

## Abstract

The Extracellular Regulated Kinase 1 and 2 transduce a variety of extracellular stimuli regulating processes as diverse as proliferation, differentiation and synaptic plasticity. Once activated in the cytoplasm, ERK1 and ERK2 translocate into the nucleus and interact with nuclear substrates to induce specific programs of gene expression. ERK1/2 share 85% of aminoacid identity and all known functional domains and thence they have been considered functionally equivalent until recent studies found that the ablation of either ERK1 or ERK2 causes dramatically different phenotypes. To search a molecular justification of this dichotomy we investigated whether the different functions of ERK1 and 2 might depend on the properties of their cytoplasmic-nuclear trafficking. Since in the nucleus ERK1/2 is predominantly inactivated, the maintenance of a constant level of nuclear activity requires continuous shuttling of activated protein from the cytoplasm. For this reason, different nuclear-cytoplasmic trafficking of ERK1 and 2 would cause a differential signalling capability. We have characterised the trafficking of fluorescently tagged ERK1 and ERK2 by means of time-lapse imaging in living cells. Surprisingly, we found that ERK1 shuttles between the nucleus and cytoplasm at a much slower rate than ERK2. This difference is caused by a domain of ERK1 located at its N-terminus since the progressive deletion of these residues converted the shuttling features of ERK1 into those of ERK2. Conversely, the fusion of this ERK1 sequence at the N-terminus of ERK2 slowed down its shuttling to a similar value found for ERK1. Finally, computational, biochemical and cellular studies indicated that the reduced nuclear shuttling of ERK1 causes a strong reduction of its nuclear phosphorylation compared to ERK2, leading to a reduced capability of ERK1 to carry proliferative signals to the nucleus. This mechanism significantly contributes to the differential ability of ERK1 and 2 to generate an overall signalling output.

## Introduction

The Extracellular-signal Regulated Kinase (ERK) cascade is a serine/threonine kinase representing the core component of a major signal transduction pathway linking cell surface to cytoplasmic and nuclear responses. ERK plays a crucial role in a wide variety of physiological processes, ranging from the control of cell proliferation to synaptic plasticity [1]–[5]. Particularly relevant is the involvement of ERK signalling in tumour development as illustrated by the high frequency of oncogenic mutations in upstream components of the cascade [6]–[8].

The ERK1/2 system is constituted by a core component represented by the Raf-MEK and ERK1/2 kinases whose activation is controlled by the Ras class of small GTPases. ERK1/2, requires being phosphorylated on both a Threonine and Tyrosine residues, thence its inactivation is provided by a class of dual specificity phosphatases that dephosphorylate both sites [9].

The two main ERK isoforms, ERK1 and 2, share approximately 85% of aminoacid identity, are activated by the same stimuli and are believed to bear similar substrate recognition properties and subcellular localization [10], [11]. Although it has been assumed that ERK1 and 2 were functionally equivalent, recent studies have shown critical functional differences between these two proteins. While the genetic ablation of ERK2 in mice results in embryonic lethality, loss of ERK1 only causes deficits in tymocyte maturation and subtle alterations in synaptic plasticity and behaviour [12], [13]. Significant differences between the two kinases also appear in the control of cell growth, in cultured fibroblasts [14], hepatocytes and liver tumor [15], [16]. One underlying functional aspect that has been elucidated relies on the differential ability of ERK1 and ERK2 to interact with the upstream MEK activator: a reduced capacity of ERK1 of interacting with MEK would decrease the signalling output of ERK1 in comparison to ERK2 [14]. Alternatively, the decreased effectiveness of ERK1 could arise further downstream on the pathway leading to nuclear signalling. Once activated in the cytoplasm, ERK1 and ERK2 translocate into the nucleus and interact with their nuclear substrates to induce specific programs of gene expression [17], [18]. Nuclear localisation of active ERK is necessary for the correct control of gene expression by growth-factors, and for morphological transformation of fibroblasts and PC12 [19]–[22]. Indeed, the speed of nucleo-cytoplasmic trafficking sets the transfer efficiency of the biochemical information carried by activated ERK to the nuclear compartment. Recently, it has been demonstrated that ERK2 continuously shuttles between the cytosolic and the nuclear compartments with a rate that depends on its phosphorylation status [23]–[25]. Since ERK2 in the nucleus is continuously dephosphorylated, the maintenance of a significant pool of active nuclear kinases requires a continuous exchange with the cytosolic fraction [24].

In the present study we demonstrate that differences in the nucleo-cytoplasmic trafficking of ERK1 and 2 significantly contribute to their differential ability to generate a signalling output. By visualizing and comparing the localisation dynamics of fluorescently tagged ERK1 and ERK2, we found that ERK1 shuttles through the nuclear membrane far more slowly than ERK2. Moreover we demonstrated that this difference is caused by a unique domain of ERK1 located at its N-terminus between residues 8–39, since the progressive deletion of this domain converts the shuttling features of ERK1 into those of ERK2. Finally, we assayed the effects of the mutated N-terminus on cell growth and demonstrated that while the deletion of the amino-terminal portion of ERK1 results in the loss of its inhibitory properties on Ras-dependent colony formation, the expression of ERK2 fused with the N-terminus of ERK1 leads to the acquisition of an ERK1-like phenotype.

## Results

### ERK1-GFP accumulates in the nucleus after stimulation

**Figure 1:**
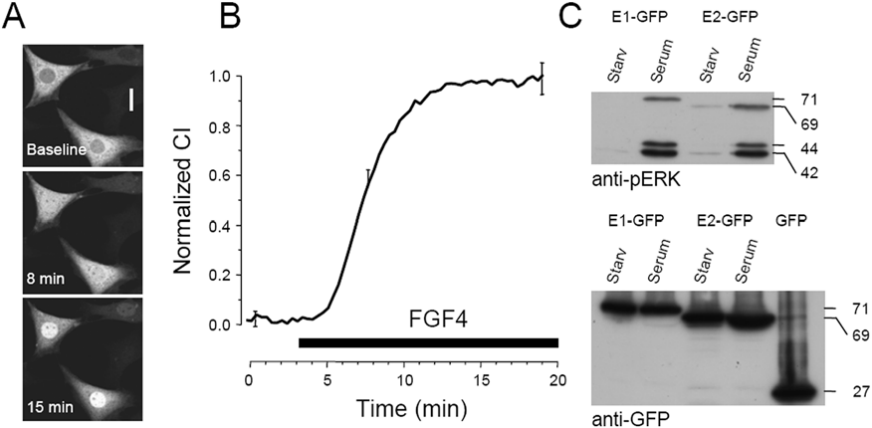
ERK1-GFP translocates in the nucleus of NIH-3T3 cells after stimulation. A) Cells transfected with ERK1-GFP in control conditions and after treatment with 80 ng/ml of FGF4 (calibration bar 20 µm). B) Time course of the normalized Concentration Index of cells stimulated with FGF4 (n = 18). The vertical bars are the standard error of the mean at the given point and are representative of the experimental variability of the entire data set. C) ERK-GFP fusion proteins are phosphorylated following serum stimulation, as demonstrated by western blot with a phospho-specific ERK antibody (upper panel). The fusion proteins have the correct molecular weight also when assayed with an anti-GFP antibody.

We initially verified the cellular localisation of ERK1-GFP fusion protein. In non stimulated cells the level of ectopic expression is crucial, since cells with very bright fluorescence invariably showed pronounced nuclear translocation, independently of ERK activation. As documented before for the ERK2-GFP fusion protein, we calibrated our imaging platforms in order to study cells where the level of expression was ≤100 nM [24]. In these conditions after 24 hr of starvation the protein localisation was cytosolic and, upon stimulation with serum (10%) or FGF4 (80 ng/ml), ERK1-GFP translocated in the nucleus (figure 1). Stimulation caused phosphorylation of the ERK-GFP fusion proteins as revealed by western blot (figure 1C): this data demonstrate that the fusion proteins can be activated, and that they are recognized both by the GFP and by the phospho-specific ERK antibodies. Furthermore, both gels show that there are no degradation products of the fusion proteins at molecular weight lower than 69/71 kDa. Identical results were obtained employing both the N-and C-terminal fusion proteins. Control cells transfected with the GFP reporter only did not display any distribution change after stimulation (data not shown).

### ERK1-GFP and ERK2-GFP shuttle across the nuclear membrane with different kinetics

**Figure 2:**
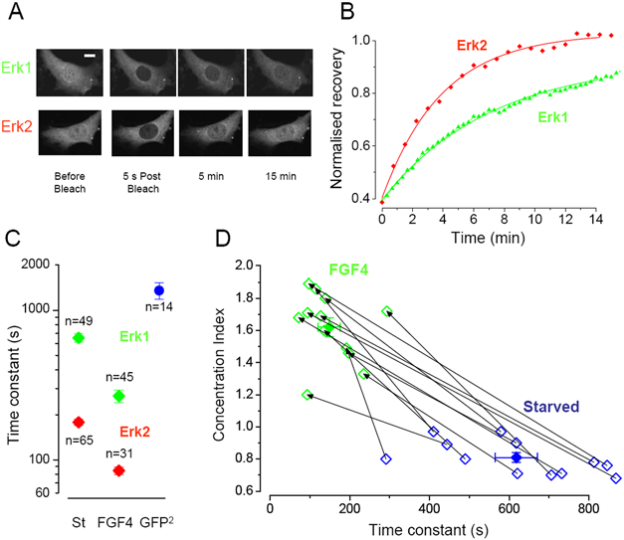
Comparison of the nucleo-cytoplasmic shuttling of ERK1 and ERK2. A) After photobleaching of the nucleus of starved NIH 3T3 cells, the ERK1 fluorescence recovered more slowly than ERK2, indicating a slower turnover of ERK1 across the nuclear membrane. Calibration bar 10 µm. B) Time course of the recovery of the cells showed in A. The data point were fitted with a single exponential with time constant τ of 235 sec (ERK2) and 430 sec (ERK1). C) Summary data for the sampled cells. The turnover of ERK1 is slower both in starved cells and after stimulation with FGF. In ERK1 starved cells the turnover is 3.7 time slower (653 s) than ERK2 (178 s) and in the stimulated cells ERK1 turnover (266 s) is 3.1 time slower than ERK2 (84 s). The blue symbol shows the turnover for a GFP dimer. This molecule is smaller than ERK-GFP but it crosses the nuclear membrane more slowly than ERK1, indicating that also for ERK1 is operating a mechanism of facilitated diffusion. D) Scatter diagram showing the recovery and Concentration Index of all paired measurements. Lines join observations relative to the same cell before and after stimulation. The filled symbols are the averages of the two groups.

ERK2 shuttles between the nuclear and cytosolic compartments with a speed that depends on its activation state. We verified whether a similar process occurs with ERK1 by photobleaching the nucleus and imaging the recovery of fluorescence [23], [24]. Since photobleaching is irreversible, the recovery in the nucleus is due to the exchange of protein between cytoplasmatic and nuclear compartments. This process is described by an exponential function with a time constant inversely proportional to the speed of permeation through the nuclear membrane. Surprisingly, we found major differences in the recovery of the fluorescence between ERK1-GFP and ERK2-GFP, indicating that ERK1 moves across the nuclear membrane at a much slower rate (figure 2). In starved fibroblasts, ERK1 turnover was 3.7 times slower than ERK2 turnover (ERK1: τ = 653 s; ERK2: τ = 178 s). After measuring the shuttling in starved cells, cultures were stimulated with FGF4 and we waited for the translocation response to reach a stable plateau before performing a new set of FRAP recordings. After activation shuttling increased for both proteins, but ERK1 still lagged behind (ERK1: τ = 266 s; ERK2: τ = 84 s). In average, shuttling accelerated with activation of a factor 2.1 for ERK2 and of a factor 2.4 for ERK1 (figure 2C). Interestingly, figure 2C shows that notwithstanding the difference with ERK2, ERK1 shuttled much faster than a GFP-GFP dimer that had a lower molecular weight (54 kD compared to 71 kD for GFP-ERK1), indicating that facilitated diffusion was occurring for ERK1 as well as for ERK2. In some cases we have measured the fluorescence recovery in the same cell before and after stimulation (figure 2D). Each cell is plotted according to the level of nuclear accumulation (see Methods) and the time constant of recovery measured in starved condition (blue symbols) and after stimulation with FGF4 (green). As shown, the speed of shuttling increased for each monitored cell. This experiment was repeated in PC12 cells differentiated with NGF to their neuron-like phenotype: here we found an even stronger difference between the two kinases (data not shown). In these cells the time constant of ERK2 shuttling varied from 610±40 s (non stimulated) to 180±15 s (stimulated, n = 17). In contrast, ERK1 shuttling varied from 780±42 to 640±46 s (n = 13). Therefore, after stimulation ERK2 was found to be 3.6 times faster than ERK1 in PC12.

### ERK-GFP fusion proteins and N-terminal mutants

**Figure 3:**
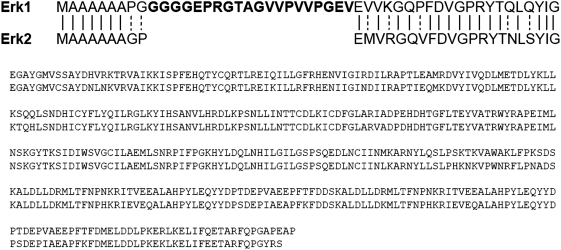
Alignment of the amino acid sequences of rat ERK1 and ERK2. The N-terminus is shown with a larger font. The 20 aa present only in ERK1 are displayed in bold.

**Figure 4:**
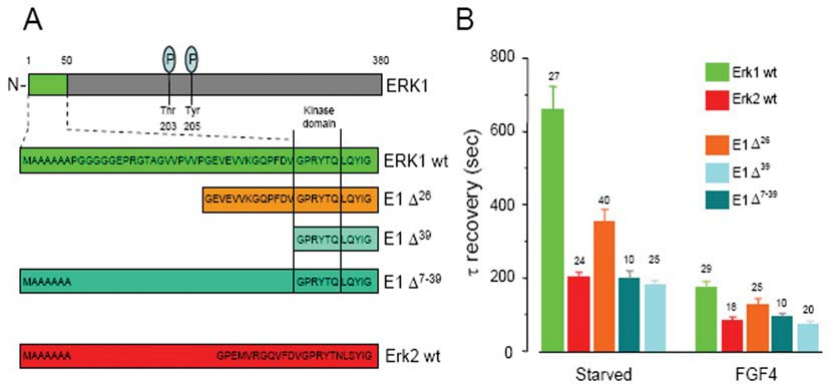
The N-terminal domain is responsible for the slow nucleo-cytoplasmic shuttling of ERK1. A) At the N-terminus, ERK1 wild type (wt) contains 20 residues not present in ERK2. We produced fusion with GFP of three different deletions of ERK1, as indicated in the diagram (mouse sequence is exemplified). B) The time constant of the nucleo-cytoplasmic shuttling of ERK1 fusion proteins is strongly affected by the different deletions of the N-terminus. In this and the next figure, the number of cells is indicated over each column.

**Figure 5:**
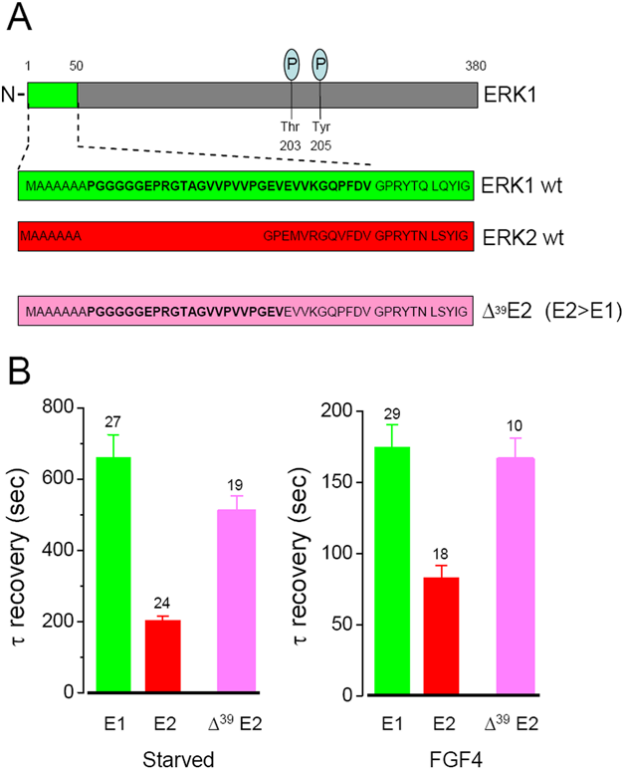
Fusion of ERK2-GFP with the N-terminus of ERK1 (Δ39 E2/E>E1). A) Alignment of the N-terminals of ERK1, ERK2 and Δ39 E2. The domain of ERK1 that has been fused to ERK2 is indicated in bold. B) The additional domain added at the N-terminus of ERK2 considerably decreases the speed of nucleo-cytoplasmic turnover both in the starved state and after stimulation with FGF4.

ERK1/2 consist of a central kinase domain flanked by short N- and C-terminal extension [26]. While activation and regulation of ERK1/2 have been extensively studied, little is known about the N-terminal region that resides outside of the kinase domain and encompasses the major sequence divergence between ERK1 and 2 (figure 3). Despite no specific functional motif has previously been mapped within this region we decided to explore the possibility that the N-terminus of ERK1 might be responsible for the altered trafficking. For this purpose we generated three fusion proteins constituted by 5′ truncated clones of ERK1, named respectively E1Δ^26^, E1Δ^39^ and E1Δ^7–39^, fused to the C-terminal GFP. As shown in figure 4A, these proteins consist of ERK1 mutants in which the N-terminus was progressively shortened, thus eliminating the major difference present at the N-terminus of ERK1 with ERK2. Symmetrically, to examine the property conferred by the residues 1–39 of ERK1 to ERK2, we generated a chimeric cDNA in which the N-terminus of ERK2 was replaced with the N-terminus of ERK1 (Δ^39^ tagged ERK2, in brief Δ^39^E2; figure 5A). Cellular localisation and FGF-induced translocation to the nucleus of these mutants was similar to what observed for the wild type ERK fusion protein (data not shown).

### The N-terminus of ERK 1 controls its nucleo-cytoplasmic shuttling

We studied the shuttling properties of the N-terminal mutants of ERK1/2 with the same FRAP protocol used above. By imaging the fluorescence recovery we found that the progressive deletion of the N-terminus of ERK1 caused an acceleration of nuclear trafficking (figure 4B). The effects of these deletions were present both in the starved and in the stimulated conditions. The shuttling time constants of the E1Δ^39^ and E1Δ^7–39^ deletion mutants were identical to what was measured for ERK2 (figure 4B). The first seven residues common to ERK1 and ERK2 do not seem to be involved in the modulation of ERK nuclear permeation since the shuttling of E1Δ^39^ and E1Δ^7–39^ were identical. Furthermore, the deletion of the initial 7 residues of ERK2 (E2Δ^7^) did not affect the shuttling rate (data not shown). These experiments indicate that the core domain that determines the difference between ERK1 and 2 are the residues 8–39. The importance of the integrity of the core domain is demonstrated by the experiment on the mutant E1Δ^26^ where the acceleration of the shuttling speed was only partial. Finally, the fusion of ERK2 with the N-terminus of ERK1 (Δ^39^E2) strongly slowed down its exchange through the nuclear membrane (figure 5B). In summary, the N-terminus domain (residues 8–39) of ERK1 appears to be necessary and sufficient to slow down the nucleo-cytoplasmic shuttling of ERK. Therefore, in terms of their trafficking properties the chimeric protein E1Δ^39^ assume the characteristics of ERK2, while Δ^39^E2 becomes like ERK1: because of the conversion of the trafficking properties of ERK1 into those of ERK2 and *viceversa*, we labelled these constructs as E1>E2 and E2>E1, respectively. At this stage two questions arise: 1) are there any functional differences between ERK1 and ERK2 that could be attributed to their trafficking differences? 2) In case that such functional difference exists, how are the signalling properties of ERK1/2 affected by modifying the N-terminus? More specifically, is E1Δ^39^ functionally equivalent to ERK2 and is Δ^39^E2 equivalent to ERK1?

### Effects of shuttling rate on ERK phosphorylation: modelling

**Figure 6:**
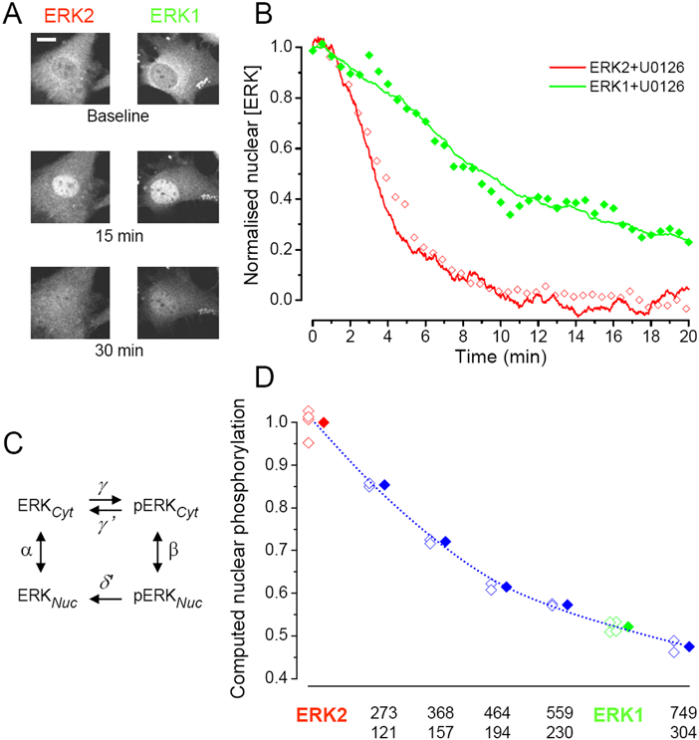
Computational estimate of the functional consequences of the different shuttling rates of ERK1 and 2. A) Cells transfected with ERK1-GFP and ERK2-GFP were treated with FGF4 for 15 min to allow complete nuclear translocation and then with the ERK blocker U0126. The inactivation of the ERK pathway caused the immediate loss of nuclear accumulation of ERK-GFP unmasking the action of nuclear dephosphorylation. Calibration bar 20 µm. B) By fitting the time course of the decay of nuclear ERK2-GFP (empty red diamonds) with the model output (continuous red line), we could estimate the dephosphorylation rate. This estimate was included in the model together with the shuttling rates measured for ERK1 to predict the outcome of this experiment practiced on the cells transfected with ERK1-GFP. The model prediction (green line) describes with great accuracy the experimental points (filled green symbols). C) Reaction scheme of the model. It has been considered an equilibrium among 4 different states regulated by first order kinetics: pERK and ERK indicates the concentration of the phosphorylated and not-phosphorylated pools in the cytoplasm (Cyt) or in the nucleus (Nuc); the rate constants α and β are the time constant of recovery of FRAP experiments; γ is the phosphorylation rate; γ' and δ' are the dephosphorylation rates, respectively, in the cytoplasm and in the nucleus. D) The model was used to compute the phosphorylation in the nucleus as a function of the shuttling speed. The empty symbols represent the result of a single simulation run and the filled symbols are the averages of each group. The phosphorylation level has been normalized to ERK2, therefore the computation shows that the total level of phosphorylation of ERK1 is only about half of ERK2. The numbers under each set of data points are the time constants (in seconds) of nuclear shuttling in the starved (above) and stimulated conditions (below).

Since ERK1/2 are activated in the cytoplasm and are continuously inactivated in the nucleus, the pool of nuclear phospho-ERK needs to be continuously replenished from the cytoplasm in order to maintain a sustained level of nuclear phosphorylation [24]. The low nuclear permeation of ERK1 suggests that during steady stimulation and translocation, a large fraction of nuclear ERK1 might be inactivated and is therefore unable to exert downstream effects. We attempted to verify this qualitative prediction by modelling ERK activation and localisation with a simple model in which ERK is considered as a first-order system equilibrating among four different states as detailed in figure 6C. Since ERK1 and 2 share the same activation mechanism, we clumped all the upstream machinery in a single first order reaction. In the diagram, pERK and ERK indicate the concentration of the phosphorylated and non-phosphorylated pools in the cytoplasm (*Cyt*) and in the nucleus (*Nuc*). When the cells are at steady state, either before stimulation or at the plateau of the translocation response, the net flux across the nuclear membrane is zero. The rates of nucleo-cytoplasmic shuttling depends on the state of phosphorylation of the protein as shown by the FRAP experiments, and α and β have been determined by the time constants of the recovery of fluorescence performed on starved and maximally stimulated cells. The remaining parameters are the rates of phosphorylation in the cytoplasm γ, and the dephosphorylation rates γ' and δ'. We assumed that the rate of phosphorylation in the nucleus is zero [27], [28], reflecting the fact that the upstream kinase MEK is actively excluded from the nucleus because of its Nuclear Exclusion Sequence [29], [30] and that its nuclear concentration is quite low [25].

To estimate the rate of dephosphorylation we have modelled data obtained previously by us [24]. Here, cells were treated with FGF4 for 15 min, until the translocation response reached plateau. Then, the MEK inhibitor U0126 was administered, causing the rapid inactivation of ERK with the consequent loss of ERK2-GFP from the nucleus. During inhibition, the phosphorylation rate drops to 0 and we can fit the model prediction of the actual time course of protein localisation to obtain an estimate of the de-phosphorylation rate. As shown in figure 6A–B the model fitted the data with great accuracy, provided that γ' = δ' = 0.003 s^−1^ corresponding to a time constant of 170 s. Then, we used the model to predict the outcome of an identical experiment performed with the ERK1 fusion protein. In this new computation we used the de-phosphorylation rate we just estimated and we inserted the translocation rates α and β measured in the FRAP experiments with the ERK1-GFP protein. Figure 6B shows that the model (continuous red line) predicted with remarkable accuracy the fluorescence loss occurring after administration of U0126. This time lapse experiment showed that ERK1 was retained in the nucleus for a much longer time than ERK2, and the modelling suggests that this was due to the lower rate of ERK1 shuttling. Next, we used the model to compute the percentage of activated ERK in the nucleus for ERK1, ERK2 and for proteins with varying shuttling rates. In this computation, we assumed that the rate of activation was identical for ERK1 and 2 and was much larger (γ = 0.02 s^−1^) than dephosphorylation [25], [31]. As shown in figure 6D nuclear phosphorylation decreased in parallel with the speed of nucleo-cytoplasmic shuttling. This indicates that the maximal level of ERK activation depends on the speed of shuttling. Interestingly, Fujioka *et al.* arrived at a similar conclusion employing an *in silico* model of the complete Ras/ERK cascade [25]. In summary modelling suggests that, even if ERK1 and 2 are subjected to identical rates of activation and inactivation, ERK2 maintains better its state of phosphorylation because of its faster nucleo-cytoplasmic shuttling.

### Effects of shuttling rate on ERK phosphorylation: biochemical evidences

**Figure 7:**
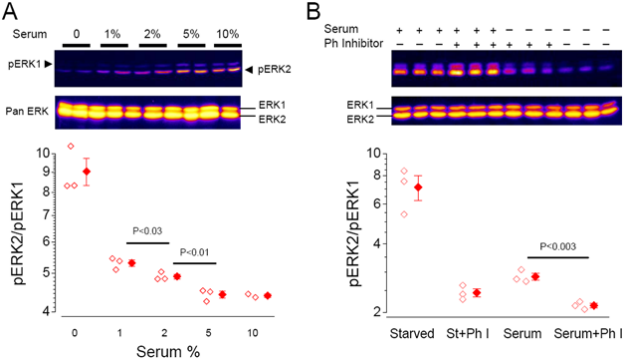
Comparison between the activation of ERK1 and 2. A) NIH 3T3 cells have been starved for 24 hr before treatment for 15 min with increasing concentrations of serum as indicated. The densitometric analysis of the gel blotted with the phospho-specific antibody has been performed with a linear imager to quantify the intensity of the phospho-ERK1 and 2 bands. From each experiment we computed the ratio pERK2/pERK1 which is a measure of the relative activation of the two kinases. The graphs shows that ERK1 activation lags behind ERK2. B) Effects of phosphatase inhibition on the relative activations of ERK1 and 2. Cells have been starved for 24 hr before a 30 min treatment with serum 10% and/or a cocktail of phosphatase inhibitors. Inhibition of phosphatases in presence of serum caused a further increase of phosphorylation compared to serum only. This increase was larger for ERK1, indicating a stronger dependence of ERK1 on de-phosphorylation. Notice that there is no vertical correspondence between the western blot and the quantification.

The results above suggested that ERK2 should activate more efficiently compared to ERK1. To verify this idea we studied the intensity/response relationship in cells treated with increasing concentrations of serum for 15 min (figure 7A). The gels have been probed with the phospho-specific ERK antibody and analyzed with a phospho-imager to ensure linearity. Since we were interested in the relative levels of activation of ERK1 and ERK2 we compared directly the phospho-specific signals rather then normalising on the total amount of protein. Therefore, the relative phosphorylation was computed as the ratio of the pERK2, pERK1 signals. If the two kinases were following the same activation profile we should expect that the ratio of activation would remain constant. On the contrary, we found that at low serum doses, ERK2 activation was larger than ERK1 indicating a better sensitivity to the stimulus. According to our model, this difference might largely be dependent on the action of nuclear phosphatases that affects more ERK1 because of its longer retention time in the nucleus. If this were true one could expect that inhibition of phosphatases would attenuate the difference in activation between ERK1 and 2. This idea was tested by immunoblotting cells which have been harvested in either starved conditions or after 30 min in 20% serum in presence or in absence of phosphatase inhibitors. As predicted, figure 7B shows that the inhibition of dephosphorylation decreased the difference between ERK1 and 2, indicating that in normal conditions ERK1 is more subjected to the action of phosphatases than ERK2. It is unlikely that this is simply due to a differential regulation of ERK1 compared to ERK2, since phosphatases have a low degree of selectivity, even more so when the targets are so similar. Interestingly, recently it has been determined that the interaction of ERK2 with dual specificity phosphatases depends on regions within the common docking domain [32], that are identical for ERK1 and ERK2. In the light of these consideration, we interpret our data as suggesting that the slower trafficking of ERK1 makes it more prone to be dephosphorylated in the nucleus. In consequence of that, ERK1 is less competent than ERK2 in activating the downstream nuclear targets.

### ERK functional efficacy depends critically on the N-terminus

**Figure 8:**
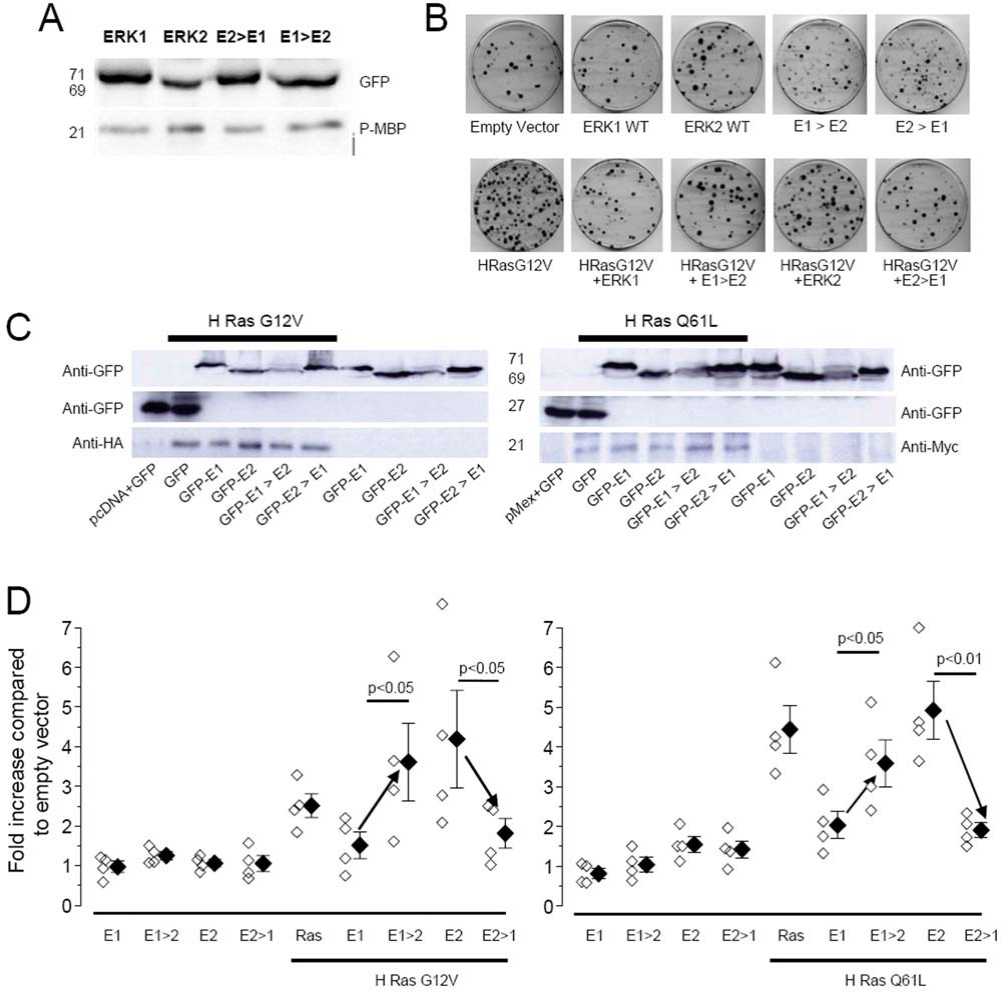
Functional consequences of N-terminus mutations of ERK1 and ERK2. A) In vitro assay testing the capacity of the indicated ERK to be activated by MEK and to phosphorylate the downstream target MBP. The upper lanes show the purified protein revealed by the anti-GFP antibody. The fusion proteins showed correct molecular weights (ERK1, E2>E1 71 kD; ERK2, E1>E2 69 kD). The lower panel indicates that each protein is able to phosphorylate MBP. The experiment was performed in triplicate with similar results. B) Representative examples of the cell colonies transfected with the indicated constructs. C) Immunoblot anti GFP shows that all the colonies expressed the GFP tagged proteins; the anti-HA and anti-Myc staining show that the mutated forms of Ras (respectively: H-Ras G12V and H-Ras Q61L), were expressed in the colonies with constitutive activation of the pathway, but they were absent in the wild type background. D) Quantification of the effect on proliferation of the various expressed fusion proteins. The symbols indicates the number of colonies counted after transfection with the specified vector, normalized to the colonies counted after transfection with the empty vector. The empty symbols represent the result of a single experiment and the filled symbols are the averages of each group. Expression of constitutively active Ras (H-Ras G12V and H-Ras Q61L) causes a large increase in proliferation that is inhibited by co-expressing ERK1 but not by co-expressing ERK2. The mutant of ERK1 characterized by fast shuttling (E1Δ39 indicated as E1>E2) did not prevent H-Ras G12V and Q61L-induced proliferation behaving similarly to ERK2. In contrast the slow mutant of ERK2 (Δ39E2 indicated as E2>E1) inhibited proliferation. Statistical significativity was essayed by *t*-test.

ERK1 and 2 have been found to have clear functional differences: while ERK2 is the most active kinase form, ERK1 appears to counteract ERK2, at least in certain cellular conditions [14]. Here we suggest that one of the possible underlying molecular causes of this difference is the slower nucleo-cytoplasmatic trafficking of ERK1 which is responsible for a deficit of ERK1 nuclear phosphorylation. Since the changes of nucleo-cytoplasmic shuttling are controlled by the N-terminus of ERK1, we asked whether the deletion of this domain (E1Δ^39^), by providing ERK1 with a faster turnover would also convert ERK1 in a kinase functionally similar to ERK2. If our prediction was right, we would also expect the reverse, i.e. a conversion of ERK2 mutant with a slow turnover (Δ^39^E2) into an ERK1-like molecule. This hypothesis was challenged with a colony formation experiment in which these ERK mutants have been tested for their effect on Ras-dependent cell growth. It has been previously demonstrated that the overexpression of ERK1 in NIH 3T3 cells inhibits growth in response to oncogenic Ras while overexpression of ERK2 does not [14]. The interactions of ERK with MEK, phosphatases and downstream targets are mediated by the common docking domain and by the catalytic domain. These domains are rather far away from the N-terminus, and so it is not expected that the modification we introduced to the N-terminus might grossly affect the activation/inactivation and catalytic properties of the fusion proteins. Nevertheless, we sought an *in vitro* indication that the GFP-tagged ERK mutants maintained catalytic activity. NIH-3T3 cells were transfected with one of four GFP-fusion proteins: ERK1-GFP, ERK2-GFP, the ERK1 N-terminus deleted (E1Δ^39^ labelled as E1>E2 in figure 8), and the fusion of the N-terminus of ERK1 with ERK2 (Δ^39^E2 labelled as E2>E1). Cells were stimulated for 15 min with 10% serum and then were prepared for protein purification (see Methods). To avoid contaminating the *in vitro* assay with the endogenous ERK1/2, we separated the GFP-tagged proteins by immunoprecipitating the cell lysates with an anti-GFP antibody. This procedure returned bands at the correct molecular weight when probed with the anti-GFP antibody (figure 8A). The immunopurified proteins were reacted in vitro with the specific ERK substrate Myelin Binding Protein (MBP) Figure 8A shows that all four fusion proteins were capable of phosphorylating MBP. If MBP was reacted in absence of purified ERK there was no signal detected by the phospho-specific MBP antibody (not shown).

To evaluate the effects of these proteins on cell proliferation, we generated colonies by transfecting NIH 3T3 with the four constructs either in presence or in the absence of two different oncogenic Ras mutants (figure 8B). Expression was controlled by western blot for the presence of the GFP-tagged constructs and for the HA tagged Ras mutants (figure 8C). The analysis of the colonies showed that on a normal background the overexpression of any chimera did not significantly modify the growth potential. In contrast, in the presence of the hyperactivated Ras mutants, the overexpression of ERK1 or ERK2 decreased or increased respectively the growth potential. This is consistent with previous results showing a competitive inhibition on proliferation by ERK1 [14]. Furthermore, the mutations of ERK1 and 2 that affect their nucleo-cytoplasmic shuttling also exert their effects on cell proliferation. Specifically, the N-terminus deletion of ERK1 (E1Δ^39^, labelled as E1>E2) did not inhibit the proliferation caused by oncogenic Ras expression. In contrast, the fusion of the N-terminus of ERK1 with ERK2 (Δ^39^E2, E2>E1) results in a molecule that inhibited Ras-mediated colony formation. Similar results where obtained transfecting constructs that, instead of carrying the GFP, were carrying a HA tag (data not shown).

**Figure 9:**
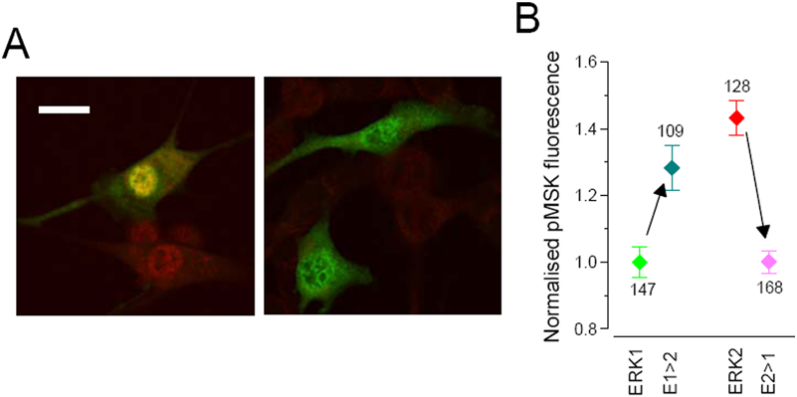
Effects of the overexpression of N-terminus mutations of ERK1/2 on the nuclear target MSK. A) Representative fields of fibroblast treated for 24 hr with 10% serum. In this example, cells have been transfected with ERK2-GFP and were stained with the pMSK specific antibody (red). The left panel show that the treatment caused nuclear translocation of ERK2-GFP and activation of MSK, which is well visible both in the transfected cell (the nucleus is yellow) and in nearby non-transfected cells. The inhibitor U0126 (25 µM) was administrated 6 hr before fixation. In these conditions ERK did not accumulate in the nucleus and there was not detectable phosphorylation of MSK, indicating that, MSK requires ERK activity to be phosphorylated after serum treatment. Bar 20 µm. B) Intensity of the pMSK signal measured in cells transfected with ERK1, ERK2, E1Δ39 (indicated as E1>E2) and Δ39E2 (indicated as E2>E1). The experiment was repeated in triplicate and the fluorescence of each cell has been normalised to the mean fluorescence of the ERK1 group to allow the comparison of the different experiments. Number of cells indicated nearby each symbol. U0126 suppressed almost completely the pMSK signal (mean = 0.48±0.04, n = 76; not shown). Since cell fluorescence was not normally distributed, significativity was assessed with the Kolmogorov-Smirnoff test. ERK1≠ERK2 (p≤0.0005); E1>E2≠E2>E1 (p≤0.0005). E1 and E2>E1 are not significantly different (p≤0.2). E1>E2 is larger that ERK1 (p≤0.0005) but it is also slightly but significantly smaller than ERK2 (p≤0.01).

A further test of the effects of the N-terminus of ERK1 on signalling to the nucleus was performed by testing the phosphorylation of a nuclear target of ERK: the downstream kinase MSK1 [33]. Cells were transfected with tagged ERK1 and 2 and the two transformed ERK1/2 (E1Δ^39^, labelled as E1>E2 and Δ^39^E2, E2>E1). Cells were cultured for 24 hours in presence of 10% serum and processed for pMSK immunofluorescence. Representative images are shown in figure 9A. On the left are shown cells transfected with ERK2-GFP: tagged ERK2 is clearly translocated into the nucleus (green), where it can also be observed a strong pMSK signal (red). The phosphorylation of MSK required ERK activation. In fact, if U0126 was added 6 hours before fixation, both GFP-ERK translocation and MSK phosphorylation were completely prevented (right panel). We measured the pMSK signal in cells expressing the tagged ERK protein from 3 separate experiments, and the cumulative results are shown in figure 9B. The overexpression of ERK1 and 2 had opposite effects on the activation of MSK. Furthermore, also the two modified proteins had opposite effects on MSK phosphorylation: the removal of the N-terminus from ERK1 favoured MSK activation, while the addition of this domain on ERK2 depressed MSK activation. This is a further indication that the N-terminal domain of ERK1 causes a change in the capability of ERK to activate nuclear targets.

## Discussion

We have studied the nucleo-cytoplasmic shuttling of ERK1 and 2 by live imaging of fluorescently tagged proteins. We found that ERK1 and 2 differ drastically in their capability of crossing the nuclear envelope and that this difference is caused by a short domain located at the N-terminus of ERK1. Since the nucleus is a site of inactivation for signalling originating at the cell membrane, the speed of permeation through the nuclear envelope is a critical determinant of the efficiency of signalling to the nucleus. We have provided computational, biochemical and functional evidence that ERK1 and 2 have different signalling capabilities. Furthermore, we have demonstrated that the N-terminus of ERK1 is necessary and sufficient to cause the differences of permeation and of functional properties between ERK1 and 2. We have showed that the rate of nucleo-cytoplasmic shuttling and its possible modulations are crucial regulators of signalling to the nucleus and represent a novel possible target for the molecular control of this pathway.

### Effects of the N-terminus of ERK1/2 on trafficking

For a long time it has been believed that ERK1 and 2 were interchangeable. This notion had originated by their vast sequence homology and by the identity of the upstream activator and of the identified substrates. Only recently it has become clear ERK1 contributes far less than ERK2 to the overall signalling output. Indeed, it has been proposed that ERK1 functions as a partial agonist of ERK2 for MEK binding, thus partially attenuating ERK2 phosphorylation and its stimulus-dependent activation [14]. However, that study did not identify the mechanisms and the domains responsible for the differences between ERK1 and ERK2. Here we believe to have not only identified one of such mechanisms but also determined the exact structural element of ERK1 which accounts for it. Most of the functional domains that have been mapped on ERK kinases, including a consensus sequence for MEK [34], [35], the catalytic domain [36] two consensus sequences for the nucleoporins [37], [38], are present on both ERK1 and 2. Thus, there are no clear reasons why the two proteins should act as differently as they do. The most evident sequence divergence is located at the N-terminus of ERK1 where a sequence of about 39 residues has not been associated previously to any specific function. We demonstrated that this sequence is necessary and sufficient to cause the difference in shuttling rates between ERK1 and 2. This has been demonstrated producing two mutants: E1Δ^39^ and Δ^39^E2. The first construct codifies for ERK1 minus its N-terminus and was found to shuttle at the same speed of ERK2: because of the conversion of its trafficking properties into those of ERK2 we labelled it as E1>E2 (figure 8, 9). Symmetrically, the addition of the N-terminus of ERK1 at the beginning of ERK2 (Δ^39^E2; labelled as E2>E1 in figure 8, 9) slowed down the trafficking to levels undistinguishable from ERK1. There are possible alternative explanations for this effect. In one scenario, the domain might interact with elements of the nuclear envelope that hinder the progression of the protein in the nuclear pore. Alternatively, the folding of the N-terminus of ERK1 might disrupt the interactions with the nucleoporins that facilitate the diffusion of ERK through the nuclear pore.

### Bidirectional communication between nucleus and cytoplasm

The nucleus represents the end station for every signalling pathway that controls gene expression in response to changes of extracellular environment. It is becoming clear that the passage of signalling molecules through the nuclear membrane requires a shuttling process that allows a steady flux of biochemical communication between nucleus and cytoplasm [39]. It is conceivable that the biological advantage of this mechanism is that, since the signalling elements are continuously moving between the two compartments, the system has a better temporal responsiveness to the external environment. Furthermore, the speed of shuttling represents a possible locus for the fine tuning of the pathway activity. When the pathway is activated, the accumulation of ERK proceeds because of a combination of factors, including increased affinity to nuclear anchors [18], [24], [40], lowered affinity to cytosolic anchors [41] and changes in the equilibrium between import and export mechanisms [29], [42]. The translocation is crucial for the signalling process: events as diverse as ERK-mediated gene expression, proliferation, differentiation and neurite extension all require the entrance of the activated protein into the nucleus [19]–[22]. ERK trafficking between cytoplasm and the nucleus occur through a variety of mechanisms, both energy dependent and independent [38], [43]–[45], but little is known on the relative contributions of these processes. The influx is mediated mainly by a process of facilitated diffusion in which ERK permeates the nuclear pore following direct interactions with domains of specific nucleoporins [37], [38], [46]. Upon stimulation, an energy dependent process might activate, possibly explaining the increased rate of shuttling [44], [45] and the shift of the equilibrium conditions of the influx/efflux toward nuclear accumulation. The efficiency of these processes is witnessed by the fact that ERK2-GFP shuttles through the nuclear membrane only marginally slower than GFP in spite of being almost three times heavier. The energy independent processes must be thermodynamically reversible. Interestingly, such a reversibility has also been suggested for energy dependent processes [43]. At steady state, either during starvation or during sustained maximal stimulation, the influx is in equilibrium with the efflux. Particularly relevant to the experiments shown in figure 6, is the identity of the system that determines the rapid exit of ERK after inactivation of MEK activity. It has been proposed that ERK is moved out of the nucleus as piggyback with MEK which, in turn, is extruded by the CRM1 mediated export [29], [30]. This system is likely to play an important role in determining ERK localization in starved cells, when the concentration of ERK is lower in the nucleus than in the cytoplasm (see figure 1A). In these conditions the flux across the membrane is small, because of the very low amount of ERK present in the nucleus and of the relatively slow rate of trafficking. However, at the peak of the response (figure 6A), both the steady state concentration of nuclear ERK and the shuttling rate are much larger so the equilibrating outward flux must be far more conspicuous. It is unlikely that the CRM1-dependent export has sufficient carrying capacity to contribute substantially to the large fluxes observed in stimulated conditions. Since the concentration of MEK in the nucleus [25], [27] is very low and one MEK molecule is required to extrude one ERK molecule, there is simply not enough MEK to bind the far more numerous ERK molecules. Indeed, the blockage of the CRM1-mediated export by leptomycin B caused little if any reduction in the rate of ERK2-GFP export after treatment with U0126 [24]. These considerations suggest that the efflux observed after blockage of MEK activation (figure 6) are due to reversible facilitated diffusion.

### Functional consequences of trafficking speed

Since the upstream kinase of ERK, MEK, is mainly localised in the cytoplasm, the maintenance of a functional level of activated ERK in the nucleus depends on the inflow of phosphorylated ERK. We speculated that the different efficacies of ERK1 and 2 might be due to the differences of their nuclear trafficking that result in a longer permanence and inactivation of ERK1 in the nucleus, as exemplified by the slow loss of nuclear accumulation following MEK blockage (figure 6). Modelling suggested that the different kinetics exhibited in this experiment by ERK2 and ERK1 can be explained by the different shuttling rates with all other factors remaining equal. The slower trafficking of ERK1 makes it more susceptible to nuclear inactivation.

A functional interpretation of the role of the N-terminus of ERK1 is that it segregates the catalytic action of ERK1 to cytoplasmic targets. Perhaps this is why two forms of ERK are present: ERK1 might control mainly the cytoplasmic targets, while only ERK2 has the capacity of interacting with both cytoplasmic and nuclear targets. Indeed, this dual action of ERK is well represented in the nervous system: here the neurotrophin and activity dependent activation of ERK1/2 is very fast occurring within minutes from stimulus onset [47], [48]. This is followed by rapid actions likely to occur on cytoplasmic targets such as the blockage of the onset of cortical LTP that occurs within minutes from ERK inhibition [49]. On the other hand the outcome of the ERK-dependent control of gene expression, which requires nuclear translocation of phosphorylated ERK, occurs later [47] suggesting that transcription-dependent processes (such as long term memory, for example) would be mainly dependent on ERK2 rather than on ERK1. Consistently with this hypothesis, recent work has demonstrated that ERK2-knockdown mice display a strong impairment of long term memory in fear conditioning, while short term memory is not affected [50]. Importantly, at least for certain forms of long-term memory, the opposite is also true, i.e. ERK1 ablation may facilitate memory formation [12]. Indeed, we might envisage a scenario in which the nuclear/cytosolic targeting of the Ras-ERK pathway is regulated by the relative concentrations of ERK1 and 2. Whether there is a developmental or activity-dependent regulation of the relative concentrations of ERK1 and 2 should be matter of future investigation.

## Materials and Methods

### Plasmid preparation

ERK1 RNA was obtained by one step RT-PCR performed with a template on 100 ng of total RNA extract from Rat brain. The forward primer was 5′-ACGTCTCGAGCGCAGTGGAG**ATG**G-3′, incorporating XhoI site (underlined) and the ERK1 ATG start codon (bold).

The reverse primer was 5′-ACGTGGATCCTGC**TTA**GGGGGCCTCTGGTGC-3′ incorporating BamHI site (underlined) and the ERK1 stop codon (bold). The amplification product was purified and cleaved with XhoI/BamHI, and ligated to the corresponding restriction site in the vector pEGFP-C2 (Clonetech, USA), to produce the fusion of GFP at the N-terminus of rat ERK1. A similar N-terminus fusion of GFP with mouse ERK1 (ATCC cat. N. 9891061) was purchased from ATCC (American Type Culture Collection). We produce a ERK1-GFP fusion at the C terminus of ERK1 by amplifying ERK1 from the ATCC plasmid (forward primer: 5′-CCGCTCGAGAGCCAAC**ATG**GCGGCGGCG-3′, incorporating XhoI site and the ERK1 ATG start codon; reverse primer: 5′-CGGGATCCGGGGCCCTCTGGCGCCC-3′ incorporating BamHI site without the ERK1 stop codon). The amplification product was purified, cleaved and ligated to the corresponding restriction site in the vector pEGFP-N3 (Clonetech, USA). Verification of correct sequence and frame was obtained from forward and reverse automated fluorescence sequencing. All crucial experiments were repeated using both the N and C terminal fusion proteins with identical results. The ERK2-GFP tagged constructs were described previously [24]. The N-terminal deleted clones and the Δ^39^E2 constructs are shown in Fig. 4 and 5 and they were obtained as follows:

**Δ^39^E2**. ERK2 mouse cDNA was amplified by PCR introducing an ApaI restriction site (underlined) upstream of the ERK2 Kinase domain:

Forward primer: 5′-AAGGGCCCGCGCTACACCACCCTCTC3′

Reverse primer: 5′CGGGATCC **TTA**AGATCTGTATCCTGGCTG3′

Underlined BamHI, in bold stop codon. The amplificate was cloned in ApaI /BamHI of rat ERK2 EGFP-C2.

**E1Δ^39^**: obtained by digesting ApaI/BamHI Rat ERK1 cDNA and cloning it in pEGFP-C2

**E1Δ^7–39^**: obtained by digesting ApaI/BamHI Rat ERK1 cDNA and cloning it in pEYFP-C1 maintaining the first seven amino acids common to the two forms of ERK

**E1Δ^26^**: obtained by digesting Sma/BamHI Rat ERK1 cDNA and cloning it in pEYFP-C1.

**E2Δ^7^**: obtained by digesting ApaI/BamHI Rat ERK2 cDNA and cloning it in pEYFP-C1

### Cell culture and transfection

NIH 3T3 cell line were cultured in Dulbecco modified medium supplemented with 10% FBS and antibiotics (100 units/ml penicillin/streptomycin). Rat pheochromocytoma PC12 cells were cultured in DMEM supplemented with 5% FBS, 10% horse serum and 1× Pen/Strep mixture in 5% CO_2_. PC12 were induced to differentiate by adding NGF to a final concentration of 50 ng/ml. The cells were plated on glass disks or on glass-bottomed dishes (Willco) at 60–70% confluence, and transfected using Lipofectamin2000 (Invitrogen) according to the manufacture's instructions. After transfection, cells were left undisturbed for 24 hr before any further experimental manipulation. Starvation was obtained by keeping the cells for 24 hours in 1% FBS. One hour before the beginning of experiment, cells were placed in a saline solution of composition: 130 mM NaCl, 3.1 mM KCl, 1.0 mM K_2_HPO_4_, 4.0 mM NaHCO_3_, 5.0 mM dextrose, 1.0 mM MgCl_2_, 2.0 mM CaCl_2_, 10 mM HEPES, 1.0 mM ascorbic acid, 0.5 mM myo-Inositol, 2 mM pyruvic acid, pH 7.3.

### Imaging

Time lapse imaging and nuclear FRAP experiments were all performed on an Olympus Fluoview 300 or on a Leica SL confocal scanning microscope equipped with high numerical aperture objectives (Olympus water immersion 60×, 0.9 NA; Leica oil immersion 63×, 1.4 NA) and Ar/K laser excitation (488 nm). Cells were firmly locked on the microscope stage and were covered with 0.7 ml of saline solution and kept at 25°C. Cells eligible for imaging had an expression of ERK-GFP lower than about 100 nM as judged by the cell fluorescence [24]. Identical rates of nuclear translocation upon stimulation were observed for both the N and C terminal fusion proteins. Control cells transfected with the reporter only did not display any distribution change. In average, the laser power employed during imaging was about 30 µW.

The Concentration Index (CI) was defined as:
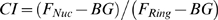
Were *BG* was the average background, *F_Nuc_* and *F_Ring_* were the average fluorescence in the nucleus and in a ring surrounding the nucleus of thickness about equal to the nucleus radius. Data plotting and statistical testing has been performed with the Origin 7 package. Details for the nuclear frap experiments have been given elsewhere [24].

### Modelling

To understand how the speed of nucleo-cytoplasmic trafficking influence the equilibrium of the activation/inactivation balance of ERK we modelled the ERK system considering that the protein equilibrates among 4 different states regulated by first order kinetics. The simulator computes the temporal evolution of the system starting from arbitrary initial conditions by means of a Montecarlo methods. In brief, the system is modelled as a collection of particles that can exists in any of the states indicates in the diagram of figure 6C. At each time point of the simulation each particle can undergo the transitions admitted by its present state or remain in the present state. The probability of each transition is given by the rate (probability per second) divided by the temporal resolution of the sequence. A random number generator is utilized to decide the particle fate. The data presented have been computed on a system with 10000 particles simulated with a time resolution of 1 s and for a duration of 5000 iterations. This time was sufficient to allow a complete evolution of the system to the equilibrium condition. Convergence to a unique solution was verified as the final state was independent on the values of the initial conditions. At least 4 simulation runs were used for each set of conditions.

### Immunoblotting

Cultures of NIH 3T3 cell lines were growth to 90% confluence in Dulbecco modified medium supplemented with 10% FBS and antibiotics (100 units/ml penicillin/streptomycin) in 60 mm Petri dishes. Before treatments the cultures were starved for 24 h in 1% serum. In some experiments cells were treated with two mixtures of phosphatases inhibitors (P2850 and P5726, Sigma Aldrich).

Cells were washed in cold PBS and lysed with 300 µl of RIPA buffer (1% Triton X-100, 0,5% Na deoxicolate, 0,1% SDS, 10% glycerol, 20 mM TrisHCL pH 8, 150 mM NaCl, 1 mM EDTA, 1 mM PMSF). Then, the samples were sonicated twice for 10 sec (Microsonics, ultrasonic cell disruptor) and boiled for 5 min in sample buffer. The same amount (around 10 µg) of cellular proteins were then subjected to SDS-PAGE in 12% gels and transferred to nitrocellulose membranes. Membranes were incubated one hour in TBS-Tween 20 containing 5% of non-fat dry milk and then exposed to a 1∶1000 dilution of rabbit polyclonal antiserum anti P-ERK (M-8159 sigma) at 4 C° over night. Membranes were washed and incubated with 1∶3000 anti-mouse IgG (H+L) conjugated to horseradish peroxidase (Bio-Rad 170-6516) for one hour at room temperature and finally revealed following the standard method for the chemiluminescence system (Bio-Rad). Gels have been exposed with the ChemiDoc analyzer and the output files were analyzed with Image J to obtain the density profiles of the bands. Quantification was performed by computing a Gaussian fit for the profiles after background subtraction. Linearity was checked by using calibration samples at known concentration of protein [24].

The same immunoblotting procedure was applied to the colonies. We employed the following antybodies: mouse monoclonal antibody anti-HA (Roche); mouse monoclonal antibody anti-c-myc (Roche). All primary antibodies were diluted 1∶1000 in blocking buffer. Membranes were washed with TBS-T and incubated with 1∶5000 peroxidase conjugated anti-mouse IgG (Amersham-Pharmacia) for one hour at room temperature and and proteins were visualized using an ECL PLUS kit (Amersham-Pharmacia) according to manufacturers' instruction.

### Immunoprecipitation, pMBP reaction and immunoblotting

NIH 3T3 cell line were cultured in 6 cm diameter petri dish at 80% of confluency and they were transfected with the vectors carrying ERK1-GFP, ERK2-GFP, E1Δ^7–39^-GFP, Δ^39^E2-GFP, by using Lipofectamine 2000 (11668-027, Invitrogen). The next day, cells were stimulated with 10% serum for 10 minutes, then they were washed in cold PBS and lysed with 0.5 ml of Triton lysing buffer (10 mM phosphate buffer, pH 7.4; 100 mM NaCl; 1% Triton X-100; 5 mM EDTA) containing 1 mM PMSF and 1 mM of phosphatase inhibitor 1 and 2 (P2850 and P5726, Sigma Aldrich). The samples were sonicated three times for 10 sec (Microsonics, ultrasonic cell disruptor) and then they were centrifuged at 4°C 10000 rpm. The supernatant were incubated 1 hour at 4°C with 50 µl of protein A-Sepharose 4B conjugate (10–1041 Zymed Laboratories, Invitogen). The samples were then centrifuged 1 minute at 700 rpm, 4°C and the supernatants were incubated with 1 µg of antibody anti-GFP (A1112, Invitrogen) on a rotating wheel at 4°C over night. The next day, 50 µl protein A-Sepharose were added to each sample on a rotating wheel 1 hour at 4°C and afterwards the samples were washed three times in 10% lysis buffer in PBS 1×.

The protein A-Sepharose conjugated with our fusion proteins were split in two aliquots for each sample, used respectively to assay the ERK1/2 phosphorylation and MBP activation.

### pMBP assay

Samples were assayed to measure the phospho-transferase activity of our fusion proteins on Myelin Binding Protein (MBP), by using the pMBP assay (17–191, Upstate). The reactions were worked out accordingly to the manufacture procedures. In brief, the immunoprecipitated samples of active ERK preparations were mixed with Mg^2+^/ATP cocktail, the Assay Diluition Buffer I (ADBI, n. cat. 20–108, Upstate) and the MAPK substrate cocktail II (n. cat. 20–166, Upstate); then, the reaction mixtures were incubated for 20–30 min in a shaking incubator kept at 30°C. Samples were then analyzed by immunoblot probed using 1 µg/ml anti-phospho-MBP, clone p12 (n. cat. 20–113, Upstate).

### Colony Formation assay in NIH 3T3

Colony Formation assay cells were performed as previously described [14], [51] with some modifications. In brief, NIH 3T3 cells were plated in 100 mm dishes, 1.5×10^5^ cells per plate and were transfected the following day. After 48 hours, cells were trypsinized and plated on 100 mm plates, 1×10^4^ cells per plate in DMEM containing 10% bovine calf serum and 0.5 mg/ml G418 (Geneticin, Gibco-Invitrogen) for the selection of neomycin-resistant cells. Each transfection sample was plated into three plates. Medium was changed every 3 days and after 10 days clones were washed with PBS and fixed in 10% formaldehyde (Sigma-Aldrich) for 15 min. Plates were then washed once with water and stained for 5 min in 0.5% crystal violet (Fluka, Sigma-Aldrich) in 20% methanol and finally washed with water to remove background staining. Images were acquired with a scanner and all colonies larger then 1.5 mm in diameter were counted.

### Immunofluorescence

Cells were washed twice with PBS, then fixed in Paraformaldehyde 4% and sucrose 4% in PBS for 20 mins. After fixation, they were rinsed twice with PBS and incubated with a blocking solution (5% FBS) and 0,2% Triton X-100 for 30 min. Afterwards the blocking solution was removed and replaced with fresh blocking containing the primary antibody (Anti-P-MSK antibody 1∶150, MBL International) and incubated overnight. The second day, cells were rinsed twice with fresh PBS and incubated for 2 hrs in a solution (1% FBS, 0,1% Triton) carrying the secondary antibody (Invitrogen, Anti-Rabbit Alexa 546, 1∶300). The cells were then rinsed twice with PBS and finally mounted with Vectashield H-1000 (Vector Labs).
